# Changing Trends in Nutritional Behavior among University Students in Greece, between 2006 and 2016

**DOI:** 10.3390/nu10010064

**Published:** 2018-01-10

**Authors:** Charikleia Kyrkou, Foteini Tsakoumaki, Maria Fotiou, Aristea Dimitropoulou, Maria Symeonidou, Georgios Menexes, Costas G. Biliaderis, Alexandra-Maria Michaelidou

**Affiliations:** 1 Department of Food Science and Technology, School of Agriculture, Faculty of Agriculture, Forestry and Natural Environment, Aristotle University of Thessaloniki, 541 24 Thessaloniki, Greece; ckyrkou@hotmail.gr (C.K.); foteinitsak@hotmail.com (F.T.); fotioum@yahoo.gr (M.F.); dimitropa@hotmail.com (A.D.); symeonidoum@yahoo.gr (M.S.); biliader@agro.auth.gr (C.G.B.); 2 Department of Field Crops and Ecology, School of Agriculture, Faculty of Agriculture, Forestry and Natural Environment, Aristotle University of Thessaloniki, 541 24 Thessaloniki, Greece; gmenexes@agro.auth.gr

**Keywords:** university students, habitual diet, gender distribution, Body Mass Index (BMI), food intake, dietary habits, Greek financial crisis

## Abstract

The objective of the present survey was to study the dietary behavior of university students residing away from the family home. In this context, we (a) compared their dietary habits in two time periods, namely 2006 and 2016; and (b) explored the possible impact of gender on the behavioral changes in nutritional choices. A total of four hundred and five university students (2006, *n* = 242; 2016, *n* = 163) participated in the study. Dietary assessment was carried out using a qualitative Food Frequency Questionnaire, while data about demographic and lifestyle factors were also collected. Students’ dietary habits have been modified in a generally desirable direction, as reflected, e.g., in the elevated consumption of several plant-based foods. Gender was also significantly associated with Body Mass Index (BMI) and changes in dietary attitudes. Possible reasons for the transition towards healthier and more balanced dietary habits could involve the budgetary constraints facing Greece in the last decade, as well as increasing nutritional awareness and other socio-cultural factors characterizing this target group. A deeper understanding of these relations would be crucial to foster nutritional education and further enhance the effectiveness of health promotion campaigns.

## 1. Introduction

Over the last few decades, there has been a steady increase in epidemiological research that provides fundamental insights into the dynamic relationship between diet, lifestyle, and health [1]. In this context, the transition from adolescence to young adulthood constitutes a stage of exceptional nutritional research interest, since university students, for the first time of their lives, often move away from the family home and as independent adults take the full responsibility for their eating habits [2,3,4,5]. However, it is reported in the literature that young adults do not have the appropriate nutritional education and experience in order to make healthy food choices [6,7], while deficient skills in meal preparation alongside the irregular and demanding class schedule have also a critical effect in the reorientation of eating habits [3,5,8]. Moreover, in most cases, students face emotional challenges and socioeconomic concerns [9,10]. Hence, attending university may lead to adoption of undesirable dietary and lifestyle patterns, that may persist throughout the future adult life [5,9,10].

Indeed, a considerable body of evidence from observational studies, conducted among youths in Western industrialized societies, has indicated a significant deterioration in the overall diet quality of this population group [2,4,6,7,9,11,12,13]. Past reports revealed that the transition to the independent living, in university, is characterized by an elevated consumption of fast food, snacks, and meat as well as a reduced intake of fruits, vegetables, and whole wheat cereal products. Furthermore, a high prevalence of other health behaviors of concern, such as smoking, excessive alcohol consumption, and physical inactivity, has been reported [14,15,16]. Similar trends were also observed among university students in developing countries [10,17,18,19,20,21]. However, to the authors’ knowledge, data regarding eating and other health-related habits of university students in countries facing budgetary constraints are rather scarce.

It is well known that, since 2009, Greece has been facing a severe debt crisis [22], which has led to a series of fiscal austerity/consolidation measures. Since then, the Greek population is being faced with a significant reduction in its overall income, resulting in a drastic downgrade of its purchasing power [23,24]. Considering the above facts, our objectives were to (a) study the dietary behavior of young university students residing away from the family home in Northern Greece by comparing their dietary habits in two time periods, namely 2006 and 2016; and (b) explore the possible impact of gender on the behavioral changes in nutritional choices in these two time periods.

## 2. Materials and Methods

### 2.1. Participants

Aristotle University of Thessaloniki (AUTh) is the largest Higher Education Institution in Greece; its mission is not limited to the production and dissemination of scientific knowledge, but it is rather expanded to the communication of messages regarding, among others, social and health related issues [25]. The public’s interest in the relationship between nutrition and health motivated us to periodically record the dietary attitudes of AUTh students, so as to explore the principles and factors that shape nutritional behavior. Through this prism, during the period between October and December 2006, undergraduate students from the Faculty of Agriculture were invited to participate in the study. Recruitment strategy included advertisements on the notice boards. In order to participate, students were required to be free of diet-related health problems. Those who were willing to participate were invited to complete a self-administered questionnaire. As such, dietary data were collected from 398 students. In 2016 (October–December), we decided to recollect dietary information, in order to explore any potential impact of financial crisis on the nutritional behavior; thus, data from 316 students were obtained.

For the present study, the exclusion process was conducted in two phases. Initially, students who lived at home with their parents or relatives (119 in 2006 and 135 in 2016) were not included, since we aimed to explore the dietary behavior of those who, for the first time, took the full responsibility for their eating habits. At a second level, those students that could not provide all the appropriate information were also excluded. Finally, a total of 405 students (242 in 2006 and 163 in 2016) were included in our analyses.

In order to carry out this survey we got the official permission from the Head of the Department of Food Science and Technology; all students volunteered to participate in the study anonymously. All participants gave their informed consent for inclusion, before they participated in the study. The procedures followed were in accordance with the guidelines laid down in the Declaration of Helsinki.

### 2.2. Data Collection

A self-administered questionnaire was used to obtain data about sociodemographic and anthropometric characteristics, lifestyle factors, and consumption frequency of selected food groups. The short version of the International Physical Activity Questionnaire (IPAQ) [26] was also used for the evaluation of the physical activity status.

Concerning sociodemographic/anthropometric characteristics and lifestyle factors, subjects were asked to provide information on gender, age, living arrangements, weight, height, and smoking behavior. Body Mass Index (BMI) was calculated as weight (in kilograms) divided by standing height (in meters squared), and BMI classification was assigned according to the World Health Organization (WHO) BMI criteria [27]. Smokers were defined as those participants who reported smoking at least one cigarette per day, while the rest of the participants were characterized as nonsmokers. Smokers were also classified in four categories, as shown in Table 1. No specific questions regarding the family or the personal income were asked.

The questionnaire incorporated a food frequency list (qualitative Food Frequency Questionnaire (QFFQ)). The QFFQ was designed to rapidly assess habitual diet. Before dietary data collection, the questionnaire was pretested to ensure the comprehensiveness and the quality of the collected information. The pilot testing was conducted, at the study site, with twenty undergraduate students and the questionnaire was revised on the basis of their feedback by a professional nutritionist. The final survey instrument—QFFQ—was composed of the following food groups: Cereals/Starchy Foods, Fruits, Νuts, Vegetables, Dairies, Meat, Processed meat, Poultry, Eggs, Fish, Legumes, Sweets, Ready-to-eat food, Olive oil, Alcoholic (i.e., wine, beer, and other alcoholic drinks) and Nonalcoholic Beverages (i.e., coffee, tea, beverages). The food groups “Cereals/Starchy Foods” and “Dairies” consisted of sub-questions regarding the type of specific food items consumed. The selection of the frequencies of consumption, shown in Table 2, was based on the traditional Mediterranean diet, as recommended by the Greek Health Authorities and represented pictorially in the Greek diet pyramid [28]. Additional questions regarding dietary behavior—i.e., meal preparation, culinary practices, number of meals per day, etc.—were also documented.

### 2.3. Methodological Strategy

**Figure 1:**
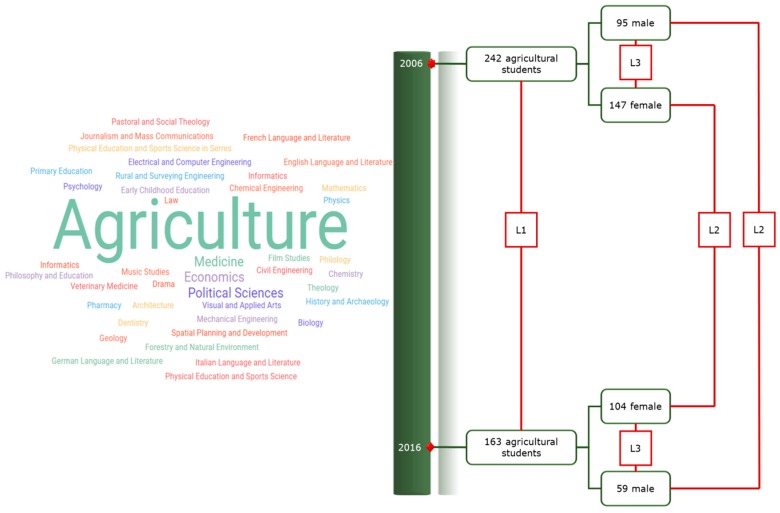
The methodological design of the study. L1: between year comparisons for the total sample; L2: between year–within gender comparisons; and L3: between gender–within year comparisons.

Our methodological approach, which is schematically outlined in Figure 1, included three levels (L) of comparison: L1, between year comparisons for the total sample; L2, between year–within gender comparisons; and L3, between gender–within year comparisons.

### 2.4. Statistical Analyses

All statistical analyses were performed with SPSS v.17.0 (SPSS Inc., Chicago, IL, USA). The significance level in all hypothesis testing procedures was predetermined at *p* ≤ 0.05. Quantitative data were presented as mean (Standard Deviation (SD)). Qualitative data were expressed as percentages (%).

Measures of skewness and kurtosis, as well as the Kolmogorov–Smirnov test were used to check for normality of the data. The Mann–Whitney U-test was used for bivariate analysis to assess differences between groups of students in quantitative variables.

Comparisons between percentages were performed by means of the Chi square test. In the Chi square tests, the observed significance level (*p* value) was computed with the Monte-Carlo simulation method utilizing 10,000 random samples [29]. The Fisher’s exact test was used for specific percentage comparisons. These two methods (Monte-Carlo simulation, Fisher’s Exact Test) lead to valid inferential conclusions even in cases where the methodological assumptions of the corresponding hypothesis testing procedures are not met. Effect sizes for significant comparisons were assessed by the value of Cramer’s V coefficient; for Cramer’s V, a value below 0.1 may be interpreted as “small”, around 0.3 as “moderate”, and 0.5 or more as “large” effect.

## 3. Results

### 3.1. Demographic/Anthropometric Characteristics

Demographic/anthropometric and selected lifestyle characteristics of the 405 university students who participated in the present study are summarized in Table 1.

Regarding BMI, the percentage of underweight students was higher in 2006 compared to 2016. Differences were also observed in the prevalence of normal weight, as the proportion of participants classified as “normal” was significantly elevated in the responders in 2016. Furthermore, the “high” physical activity level was reported by an increased proportion of young adults in 2016.

**Figure 2:**
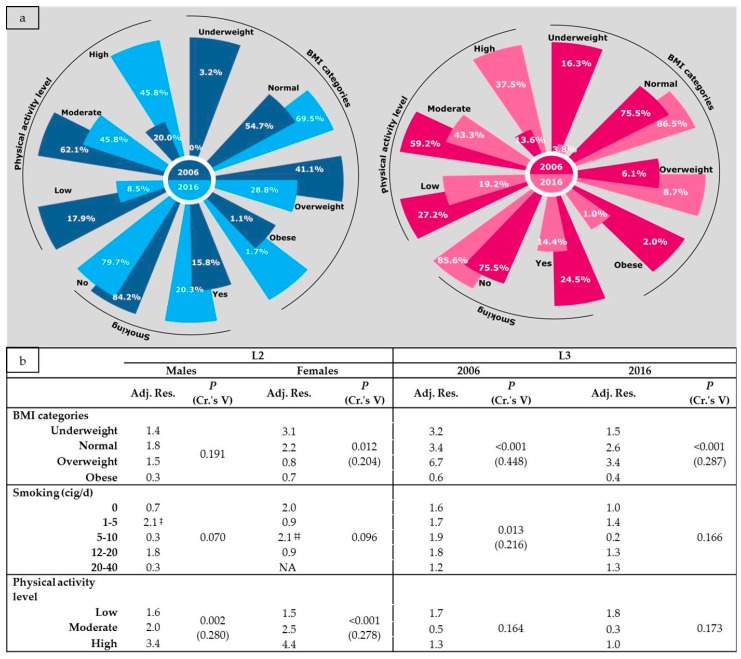
Distribution (**a**) and comparisons (**b**) of participant’s characteristics according to gender and year. BMI: Body Mass Index; L2: between year–within gender comparisons; L3: between gender–within year comparisons; Adj. Res.: Adjusted Residual (in absolute value); *P*: Chi square test *p*-value; Cr.’s V: Cramer’s V coefficient; ^‡^ Exact probability *p*-value = 0.033; ^‡‡^ Exact probability *p*-value = 0.032.

Participants’ characteristics according to gender and year are depicted in Figure 2. Between year–within gender comparisons (L2) demonstrated (i) a statistically significant increase in the predominance of normal-weight individuals among female students (75.5% vs. 86.5%) and (ii) a significant decrease in the prevalence of underweight only among females (16.3% vs. 3.8%). When within year–between gender comparisons of BMI category were conducted (L3), several statistically significant differences were also unveiled. In 2006, females were more likely to be either underweight (16.3% vs. 3.2%) or of normal weight (75.5% vs. 54.7%) compared to male students. Moreover, males were almost seven times more likely to be overweight (41.1%) compared to females (6.1%). Similar trends, concerning only normal and overweight BMI category, were also observed for participants in 2016 (Figure 2). However, in case of overweight BMI category, the observed between gender–within year differences were less pronounced, as males were almost three times more likely to be overweight (28.8%) compared to females (8.7%).

### 3.2. Dietary Characteristics

Dietary characteristics of the 405 participants, according to gender and year are summarized in Table 2, as well as in Figure S1. When participants’ dietary behavior between the two time periods were compared (L1), several statistically significant differences were noted. However, we discuss those differences with the higher effect sizes (Cramer’s V coefficient), as well as the higher adjusted residuals (>2.0 in absolute value), as the latter indicate major influences on the significance of Chi-square statistic. In particular, in 2016, elevated consumption of several plant-based foods was recorded (Table 2). One of the most striking differences observed was the increased percentage of university students reporting consumption of cereals/starchy foods more than five times per day that is 74.8%, in contrast to 24.4% in 2006. Fruit consumption also increased, as the percentage of students who consumed fruits less than one per day dropped out from 10.3% in 2006 to 0.0% in 2016; a similar, but more pronounced, trend was recorded for vegetables (Table 2). As for vegetables, another point that merits reporting is the increased percentage of participants consuming four portions per day (27.6% in 2016 vs. 1.2% in 2006). Year-related differences were also found for nuts, as the decrease in occasionally consumption was followed by an increase in weekly and daily consumption (Table 2).

Regarding the main dishes, it should be highlighted that the percentage of students choosing meat less than once per month raised from 2.5% in 2006 to 18.4% in 2016. Another observation that deserves attention is the decreased meat consumption reported in 2016 for the frequency two to three times per week, which was accompanied by an increase in fish and legumes consumption (Table 2). At this point we speculate that the increased percentage of participants consuming more than two dairy products per day (59.5% in 2016 vs. 27.3% in 2006) may ensue from the current trend to enjoy, as part of quick and easy meals, sandwiches with cheese; this speculation is further supported by the fact that the consumption of processed meat, including in our study ham and turkey, was also comparatively elevated (Table 2).

Regarding the comparisons within gender between the two time periods (L2), female students presented a greater upward tendency in cereals/starchy foods consumption (9.7 adjusted residual in absolute value). In particular, 73.1% in 2016 vs. 12.9% in 2006 consumed cereals/starchy foods more than five times per day, indicating that women are predominantly responsible for the drastic increase in cereals/starchy foods consumed in 2016 (Table 2). Concerning nuts, the increase in weekly intake (two to three per week) was more pronounced among women (27.2% in 2006 vs. 57.7% in 2016), while that of daily consumption among men (4.2% in 2006 vs. 18.6% in 2016). Respectively, the upward tendency in dairy products consumption seems to be more marked among men, who are the main drivers in the overconsumption of this food group, taking into account that the recommended intake for this population group is two portions per day. Similarly, in terms of fish, men showed approximately more than a double rise in the frequency “two to three per week” (44.2% in 2006 vs. 79.7% in 2016) compared to women (60.5% in 2006 vs. 76.0% in 2016).

As far as the between gender–within year comparisons are concerned (L3), the most pronounced observations refer to the consumption of cereals/starchy foods and fish. Specifically, in 2006, 42.1% of men consumed cereals/starchy foods more than five times per day compared to 12.9% of women. On the contrary, women in 2006 consumed more frequently fish, i.e., 60.5% of women compared to 44.2% of men. These between-gender differences were alleviated in 2016.

## 4. Discussion

The present study was carried out in order to explore the potential changes in dietary behavior among Greek university students, moving away from the family home, in two time periods, namely 2006 and 2016, and to determine possible gender-related nutritional trends. The main findings could be summarized as follows: (a) most of the respondents in our study belonged to the normal BMI category, followed by overweight young male adults in both time periods; and (b) young Greek adults modified their dietary habits in a generally desirable direction.

Although the vast majority of the participants, in both time periods, were of normal BMI, a higher percentage, namely 80.4%, was recorded in 2016. A possible explanation could be the transition towards healthier and more balanced dietary choices along with the higher rates of physical activity recorded in 2016, highlighting the importance of physical activity status as a modifiable lifestyle factor [2,30,31]. Nevertheless, the consistently higher percentage of overweight among males compared to females is a cause of concern. This gender-specific difference has been reported in other studies as well [2,13,32,33] and may be partly attributed to alcohol consumption, which, according to French et al. (2010) [34], is a possible risk factor for weight gain. However, it would be an oversimplification to ascribe this observation to a particular dietary choice. Thus, it is tempting to hypothesize that this continuous trend may be linked to the gender-related perception of portion size [35,36,37]. Indeed, it is reported in the literature that men tend to consume larger portions compared to women [35,36,37]. Furthermore, according to Ansari et al. (2010) [38], “men find a greater variety of body shapes to be socially acceptable than women, whereas women have a narrower range of what is considered to be the ‘ideal’ body image”. Consequently, women are more prone to dieting [39], in order to fulfill their desire to be attractive [31,40].

Concerning dietary data, the study findings document a shift towards positive dietary changes between the two time periods. Within this context, students in 2016 presented greater adherence to the national and global dietary recommendations [41,42], since 74.8% of the participants consumed cereals/starchy foods—the key energy suppliers—more than five times per day. Furthermore, in 2016 a higher proportion of the participants followed the guidelines [41,42] for vegetable and fruit consumption. At a first glance, these findings seem to contradict previous literature information suggesting that poor financial resources (low income) are associated with inadequate intake of fruit, vegetables, and other nutritious foods [43,44,45,46]. However, aspects of this trend do not apply for Greece, which ranks among the major producers of fruit and vegetables in the European Union [47]. Thus, low price, high quality and increased availability of fresh, seasonal produce may possibly explain the increase in their consumption. At this point, it is worth mentioning that the last decades have witnessed a surging interest by the academic community in the protective effects of plant based products, which are probably mediated through multiple beneficial nutrients contained in these foods [48]. Along with macronutrients, plant foods contain appreciable amounts of some vitamins and minerals as well as dietary fiber [48]. Being the cornerstones of the traditional Mediterranean diet, products of plant origin are allies for a healthy lifestyle [48].

Furthermore, according to the Mediterranean mentality, legumes and small, cheap fish, such as sardines, do represent traditional dishes. Hence, the relatively increased consumption of these products, as recorded in the present study, was followed by a decrease in meat consumption. Similar downward tendencies were also noted in terms of “ready-to-eat food”, suggesting that the budgetary constraints of the last decade encourage young adults to dedicate more time to meal preparation at home, even in the form of quick and easy meals.

Moreover, the notable enhancement in diet quality may be also attributed to the increased awareness between diet and health [49,50]. Specifically, in view of the general consensus that dietary habits are of importance to the etiology of several chronic diseases [51], educational institutions, mass media, and health-promotion organizations have designed campaigns to convey messages on the health promoting dimensions of a balanced diet.

However, our study has some potential limitations when considering the generalizability of the findings. At first, the possibility of selection bias cannot be excluded as students participated on a voluntary basis. Secondly, the respondents were enrolled in the Agricultural Science Faculty and, therefore, might have greater nutritional or food-related background knowledge. Further, all data were obtained by self-reporting, and may be biased by the limitations of memory or by the desire to follow social expectations. Concerning the possible impact of financial constraints on the behavioral changes, the authors did not gather any data on the financial status of the participants for the following reasons: (a) in 2006 Greece was not affected by financial crisis and consequently financial constraints have not been considered as a potential factor explaining differences in dietary choices; and (b) people are often unaware of household income or reluctant to provide such information that is usually believed to be a rather sensitive issue to respond to [50]. Finally, as in any cross-sectional observational study, no causal relationships can be drawn [52,53].

Despite the above limitations, there are a number of strengths that should be realized when interpreting the results of the present work. To our knowledge, this is the first study attempting to investigate trends in dietary behavior, involving students as the target group, and following the economic downturn started in Greece in 2009. University students represent, according to the literature [3], a target group with minimal variability in respect to socio-demographic and lifestyle factors such as age, health and education compared to a population-based sample. In this context, they comprise a suitable sample for examining dietary habits [54]. Furthermore, the anonymous, brief frequency and without portion sizes questionnaire used in the present survey may further encourage reliable recording from respondents. It should also be highlighted that the distributed questionnaire was self-administered, thus requiring limited resources.

## 5. Conclusions

It is reported in the literature [2,4,6,7,9,11,12,13] that the transition from adolescence to independent life is, in most cases, characterized by a significant deterioration in the overall diet quality. This phenomenon might be also influenced by financial constraints of the individuals involved. However, the results of the present survey send a hopeful message for potential health benefits regarding the changes of the dietary profile among university students over the last ten years. Several factors might be relevant for the observed dietary patterns. It seems that the potential financial constraints have provided an incentive towards “healthier” lifestyle and food choices, such as meal preparation at home and higher vegetable and fruit consumption. Furthermore, we cannot rule out the possibility that these positive nutritional behaviors may have ensued from the increasing awareness on the lifelong relation between diet and health; this could, in turn, be attributed to the improved nutritional education [49,50]. Overall, it is of utmost importance to realize that messages promoting the health benefits of certain dietary patterns, without considering other influential factors, such as economic constraints and cultural aspects, are unlikely to be successful [55]. Thus, nutrition educational programs for university students should rather focus on increasing their knowledge-awareness and skills in improving dietary quality in an affordable way. Understanding the interrelations among health, dietary quality, and food cost would be crucial in order to enhance the effectiveness of any health promotion campaigns launched for this target group.

## Figures and Tables

**Table 1:** Demographic/anthropometric and selected lifestyle characteristics of the 405 students who participated in the study.

	2006	2016		
	Mean (SD)	Mean (SD)		*P* ^1^
Age (year)	21.07 (1.66)	20.98 (1.38)		0.739
	**L1**			
	**%**	**%**	**Adj. Res.**	***P*** **^2^ (Cr.’s V)**
Gender				
Men	39.3	36.2	0.6	0.601
Women	60.7	63.8	0.6	
BMI categories				
Underweight	11.2	2.5	3.2	0.004 (0.178)
Normal	67.4	80.4	2.9	
Overweight	19.8	16.0	1.0	
Obese	1.7	1.2	0.3	
Smoking (cig/d)				
0	78.9	83.4	1.1	0.299
1–5	6.6	8.0	0.5	
5–10	11.2	5.5	2.0 ^‡^	
12–20	2.9	1.8	0.7	
20–40	0.4	0.6	0.3	
Physical activity level				
Low	23.6	15.3	2.0	<0.001 (0.273)
Moderate	60.3	44.2	3.2	
High	16.1	40.5	5.5	

SD: Standard Deviation; BMI: Body Mass Index; L1: between year comparisons for the total sample; Adj. Res.: Adjusted Residual (in absolute value); *P* ^1^: *p*-value as derived by Mann–Whitney U test; *P* ^2^: Chi square test *p*-value; Cr.’s V: Cramer’s V coefficient; ^‡^ Exact probability *p*-value = 0.050.

**Table 2:** Dietary characteristics of the 405 participants according to gender and year.

	L1									L2				L3			
						2006		2016		Within Male Students		Within Female Students		Between Gender in 2006		Between Gender in 2016	
		2006	2016			Male	Female	Male	Female								
		%	%	Adj. Res.	*P* (Cr.’s V)	%	%	%	%	Adj. Res.	*P* (Cr.’s V)	Adj. Res.	*P* (Cr.’s V)	Adj. Res.	*P* (Cr.’s V)	Adj. Res.	*P* (Cr.’s V)
Cereals/Starchy foods	1/day	1.2	0.6	0.6	<0.001 (0.510)	0.0	2.0	0.0	1.0	NA	<0.001 (0.373)	0.7	<0.001 (0.627)	1.4	<0.001 (0.364)	0.8	0.802
	2/day	8.3	0.0	3.8		8.4	8.2	0.0	0.0	2.3		3.0		0.1		NA	
	3/day	21.9	6.7	4.1		11.6	28.6	8.5	5.8	0.6		4.5		3.1		0.7	
	4/day	24.0	6.7	4.5		17.9	27.9	5.1	7.7	2.3		4.0		1.8		0.6	
	5/day	20.2	11.0	2.4		20.0	20.4	8.5	12.5	1.9		1.6		0.1		0.8	
	>5/day	24.4	74.8	10.0		42.1	12.9	78.0	73.1	4.4		9.7		5.2		0.7	
Fruits	<1/day	10.3	0.0	4.2	<0.001 (0.258)	9.5	10.9	0.0	0.0	2.4	0.049 (0.250)	3.5	<0.001 (0.276)	0.4	0.630	NA	0.904
	1/day	25.2	17.8	1.8		24.2	25.9	16.9	18.3	1.1		1.4		0.3		0.2	
	2/day	35.1	42.3	1.5		41.1	31.3	42.4	42.3	0.2		1.8		1.6		0.0	
	3/day	16.1	16.0	0.0		13.7	17.7	18.6	14.4	0.8		0.7		0.8		0.7	
	>3/day	13.2	23.9	2.8		11.6	14.3	22.0	25.0	1.7		2.1		0.6		0.4	
Nuts	<1/month	20.2	18.4	0.5	<0.001 (0.337)	13.7	24.5	23.7	15.4	1.6	<0.001 (0.387)	1.8	<0.001 (0.367)	2.0	0.012 (0.213)	1.3	0.021 (0.242)
	O	42.6	14.7	5.9		36.8	46.3	6.8	19.2	4.2		4.4		1.4		2.2	
	2–3/week	34.3	55.2	4.2		45.3	27.2	50.8	57.7	0.7		4.9		2.9		0.8	
	D	2.9	11.7	3.5		4.2	2.0	18.6	7.7	2.9		2.2		1.0		2.1	
Vegetables	<1/day	21.1	0.0	6.3	<0.001 (0.600)	33.7	12.9	0.0	0.0	5.0	<0.001 (0.663)	3.8	<0.001 (0.563)	3.9	0.002 (0.272)	NA	0.031 (0.233)
	1/day	53.7	25.2	5.7		49.5	56.5	30.5	22.1	2.3		5.4		1.1		1.2	
	2/day	19.4	40.5	4.6		13.7	23.1	28.8	47.1	2.3		4.0		1.8		2.3	
	3/day	4.1	0.0	2.6		3.2	4.8	0.0	0.0	1.4		2.3		0.6		NA	
	4/day	1.2	27.6	8.1		0.0	2.0	37.3	22.1	6.4		5.1		1.4		2.1	
	>4/day	0.4	6.7	3.7		0.0	0.7	3.4	8.7	1.8		3.2		0.8		1.3	
Dairies	<1/day	2.1	0.6	1.2	<0.001 (0.325)	1.1	2.7	0.0	1.0	0.8	<0.001 (0.466)	1.0	0.001 (0.253)	0.9	0.529	0.8	0.065
	1/day	28.1	14.1	3.3		27.4	28.6	13.6	14.4	2.0		2.6		0.2		0.2	
	2/day	42.6	25.8	3.5		47.4	39.5	15.3	31.7	4.1		1.3		1.2		2.3 ^‡‡^	
	>2/day	27.3	59.5	6.5		24.2	29.3	71.2	52.9	5.7		3.8		0.9		2.3 ^‡‡‡^	
Meat	<1/month	2.5	18.4	5.5	<0.001 (0.420)	1.1	3.4	18.6	18.3	4.0	<0.001 (0.400)	3.9	0.000 (0.441)	1.1	0.328	0.1	0.377
	O	22.7	46.6	5.0		24.2	21.8	40.7	50.0	2.2		4.7		0.4		1.1	
	2–3/week	72.3	34.4	7.6		70.5	73.5	39.0	31.7	3.9		6.6		0.5		0.9	
	D	2.5	0.6	1.4		4.2	1.4	1.7	0.0	0.9		1.2		1.4		1.3	
Processed meat	<1/month	11.2	11.0	0.0	<0.001 (0.207)	7.4	13.6	10.2	11.5	0.6	0.182	0.5	0.002 (0.240)	1.5	0.078	0.3	0.876
	O	28.9	17.8	2.6		24.2	32.0	15.3	19.2	1.3		2.2		1.3		0.6	
	2–3/week	54.5	54.6	0.0		60.0	51.0	55.9	53.8	0.5		0.4		1.4		0.3	
	D	5.4	16.6	3.7		8.4	3.4	18.6	15.4	1.9		3.4		1.7		0.5	
Poultry	<1/month	0.8	5.5	2.9	0.003 (0.181)	2.1	0.0	6.8	4.8	1.5	0.345	2.7	0.003 (0.216)	1.8	0.051	0.5	0.929
	O	23.6	20.2	0.8		27.4	21.1	22.0	19.2	0.7		0.4		1.1		0.4	
	2–3/week	75.2	71.2	0.9		69.5	78.9	67.8	73.1	0.2		1.1		1.7		0.7	
	D	0.4	3.1	2.2		1.1	0.0	3.4	2.9	1.0		2.1		1.2		0.2	
Eggs	<1/month	14.0	26.4	3.1	<0.001 (0.289)	10.5	16.3	18.6	30.8	1.4	0.002 (0.304)	2.7	<0.001 (0.286)	1.3	0.089	1.7	0.065
	O	41.3	19.0	4.7		37.9	43.5	15.3	21.2	3.0		3.7		0.9		0.9	
	2–3/week	43.8	47.9	0.8		49.5	40.1	54.2	44.2	0.6		0.6		1.4		1.2	
	D	0.8	6.7	3.3		2.1	0.0	11.9	3.8	2.5		2.4		1.8		2.0	
Fish	<1/month	6.6	12.3	2.0	<0.001 (0.318)	5.3	7.5	11.9	12.5	1.5	<0.001 (0.431)	1.3	<0.001 (0.241)	0.7	0.014 (0.186)	0.1	0.818
	O	39.3	10.4	6.4		50.5	32.0	8.5	11.5	5.3		3.8		2.9		0.6	
	2–3/week	54.1	77.3	4.7		44.2	60.5	79.7	76.0	4.3		2.6		2.5		0.5	
Legumes	<1/month	6.6	2.5	1.9	<0.001 (0.336)	7.4	6.1	1.7	2.9	1.5	<0.001 (0.330)	1.2	<0.001 (0.341)	0.4	0.955	0.5	0.908
	O	33.9	7.4	6.2		33.7	34.0	8.5	6.7	3.6		5.1		0.1		0.4	
	2–3/week	59.5	90.2	6.7		58.9	59.9	89.8	90.4	4.1		5.3		0.1		0.1	
Sweets	<1/month	5.8	2.5	1.6	0.259	11.6	2.0	0.0	3.8	2.7	0.036 (0.236)	0.9	0.722	3.1	0.004 (0.233)	1.5	0.221
	O	16.5	16.6	0.0		21.1	13.6	22.0	13.5	0.1		0.0		1.5		1.4	
	2–3/week	54.5	51.5	0.6		47.4	59.2	47.5	53.8	0.0		0.8		1.8		0.8	
	D	23.1	29.4	1.4		20.0	25.2	30.5	28.8	1.5		0.6		0.9		0.2	
“Ready-to-eat food”	<1/month	5.0	7.4	1.0	0.016 (0.157)	2.1	6.8	6.8	7.7	1.5	0.069	0.3	0.269	1.6	0.035 (0.186)	0.2	0.707
	O	28.1	22.1	1.4		23.2	31.3	18.6	24.0	0.7		1.3		1.4		0.8	
	2–3/week	62.8	70.6	1.6		67.4	59.9	74.6	68.3	1.0		1.4		1.2		0.8	
	D	4.1	0.0	2.6		7.4	2.0	0.0	0.0	2.1		1.5		2.0		NA	
Alcoholic beverages	<1/month	7.4	3.7	1.6	0.228	4.2	9.5	5.1	2.9	0.3	0.695	2.1 ^‡^	0.138	1.5	0.006 (0.223)	0.7	0.016 (0.247)
	O	25.6	24.5	0.2		18.9	29.9	13.6	30.8	0.9		0.1		1.9		2.5	
	2–3/week	62.0	63.2	0.2		67.4	58.5	66.1	61.5	0.2		0.5		1.4		0.6	
	D	5.0	8.6	1.5		9.5	2.0	15.3	4.8	1.1		1.2		2.6		2.3	

L1: between year comparisons for the total sample; L2: between year–within gender comparisons; L3: between gender–within year comparisons; Adj. Res.: Adjusted Residual (in absolute value); *P*: Chi square test *p*-value; Cr.’s V: Cramer’s V coefficient; O: occasionally; D: daily; NA: not applicable; ^‡^ Exact probability *p*-value = 0.039; ^‡‡^ Exact probability *p*-value = 0.021; ^‡‡‡^ Exact probability *p*-value = 0.022.

## Supplementary Materials

The following are available online at http://www.mdpi.com/2072-6643/10/1/64/s1. Figure S1, Dietary habits of male and female students in 2006 (men: *n* = 95, women: *n* = 147) and 2016 (men: *n* = 59, women: *n* = 104).
