# Supplementary figures and images for: Changing Trends in Nutritional Behavior among University Students in Greece, between 2006 and 2016
Source: Nutrients. 2018 Jan 10;10(1):64. doi: 10.3390/nu10010064 (PMC5793292; doi:10.3390/nu10010064)

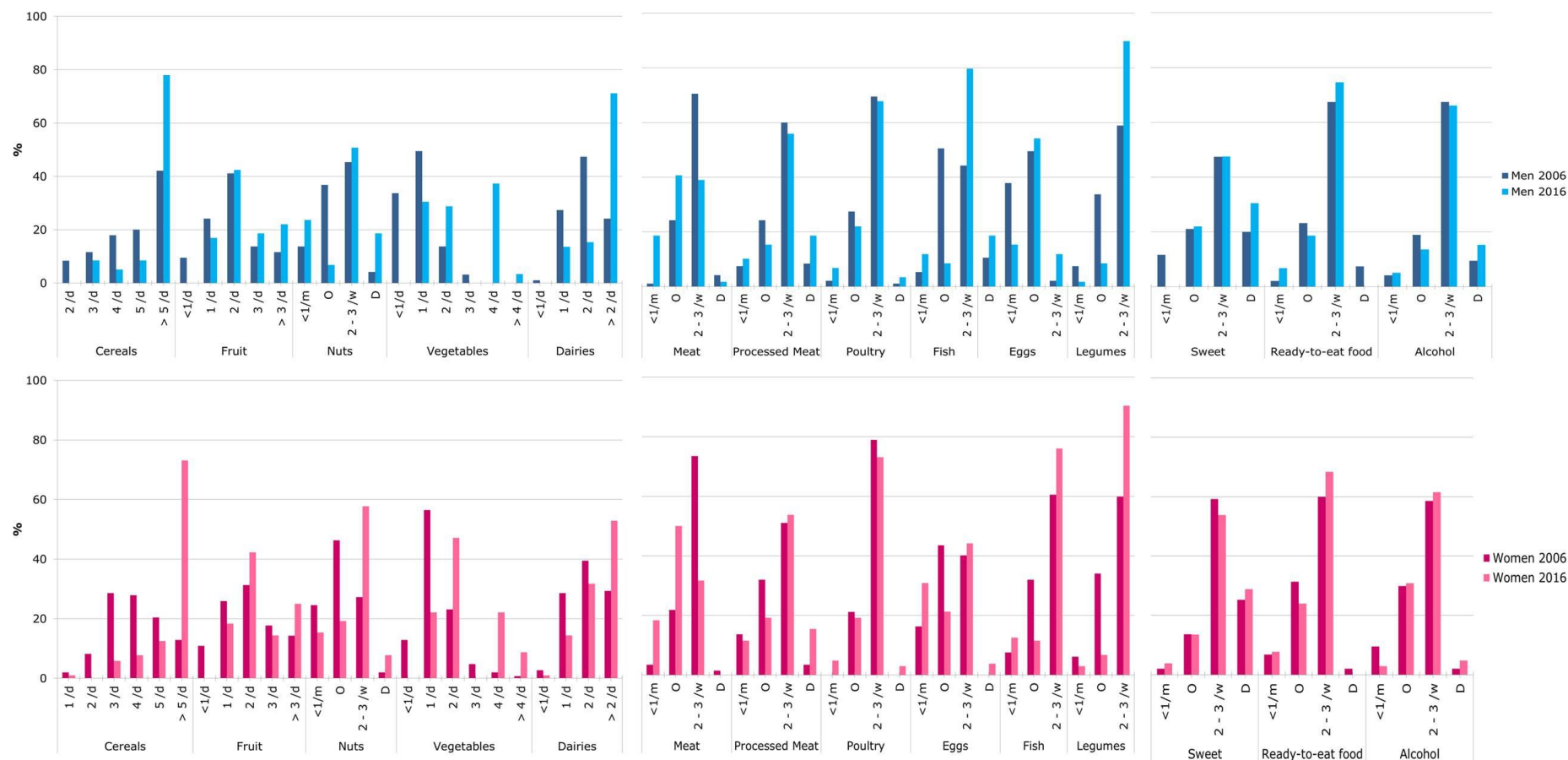

Figure S1: Dietary habits of male and female students in 2006 (men:  $n = 95$ , women:  $n = 147$ ) and 2016 (men:  $n = 59$ , women:  $n = 104$ ).

Supplement: Supplementary file 1 [file nutrients-10-00064-s001.pdf]
